# A Targeted Nanoprobe Based on Carbon Nanotubes-Natural Biopolymer Chitosan Composites

**DOI:** 10.3390/nano6110216

**Published:** 2016-11-17

**Authors:** Baoyan Wu, Na Zhao

**Affiliations:** MOE Key Laboratory of Laser Life Science & Institute of Laser Life Science, College of Biophotonics, South China Normal University, Guangzhou 510631, China; Zhaona5251@163.com

**Keywords:** carbon nanotubes, chitosan, targeting, imaging, photodynamic therapy

## Abstract

A novel targeting theranostic nanoprobe based on single-walled carbon nanotubes (SWCNTs)-natural biopolymer chitosan composites was developed for cancer cell targeting imaging and fluorescence imaging-guided photodynamic therapy. First, chitosan was respectively conjugated with a tumor-homing molecule folic acid, or a photosensitizing drug pyropheophorbide a using a water-soluble carbodiimide coupling chemistry. Chitosan was fluorescently labeled by fluorescein isothiocyanate via the covalently linkage of the isothiocyanate group with the amino group. Second, SWCNTs were sonicated in the functional chitosan aqueous solution for 6 h at room temperature in order to obtain the nanoprobe (PPa/FITC-SWCNT-FA). The as-prepared nanoprobe has been characterized with transmission electron microscope, confocal microscopy, and cell cytotoxicity tests. Chitosan was decorated onto SWCNTs resulting in the water-dispersible PPa/FITC-SWCNT-FA, and can be selectively transported inside folate receptor-positive tumor cell with good targeting imaging. PPa/FITC-SWCNT-FA exhibited low dark toxicity about 7%–13%, and high phototoxicity about 60%–74% against HeLa cells upon a 635 nm laser irradiation, indicating satisfying biocompatibility and antitumor activity. These results suggest the study could offer a feasible alternative to presently available nanoparticle-based theranostic agents.

## 1. Introduction

Photodynamic therapy (PDT) is an alternative tumor-ablative and function-sparing oncologic intervention [1]. PDT is a clinically approved, minimally invasive therapeutic procedure mediated by the production of singlet oxygen or reactive oxygen species generated from photosensitizer molecules with an appropriate excitation light to cause cell death and tissue destruction [2,3,4]. Compared to conventional cancer therapy, the major advantages of PDT are that a photosensitizer itself is of low toxicity in the dark, and activation by light alone permits minimal damage to non-specific tissues [5,6,7]. Furthermore, the activating light irradiation does not directly damage, so its effect on tissues or neighboring cells without the photosensitizing drug is not harmful [8,9].

PDT has emerged as a disease site specific treatment modality [1]. Unfortunately, most photosensitizers used clinically are hydrophobic and low solubility in physiological media [10]. Hydrophobic nature of most photosensitizers results in strong self-aggregation in aqueous media, which significantly reduces their photodynamic efficacy because only monomeric species are appreciably photoactive [11]. Various nanoparticle-based photosensitizers have been synthesized, such as a cerasomal photosensitizer [12], chlorin e6-conjugated silica-coated gold nanoclusters [13], microneedles, and gold nanoparticle-based PDT [14,15]. Photosensitizer-carrying nanoparticles could increase the water solubility of photosensitizer molecules, enhance their tumor accumulation, and thus improve the therapeutic efficacy and specificity of PDT [6].

Carbon nanotubes are carbon clusters (polymers) with huge molecular weight and have been the central material in the field of nanomaterials science and nanotechnology [16], which has been used for biological applications such as biosensors and targeted delivery of drugs [17,18,19,20]. Carbon nanotubes are less invasive to living organisms than conventional approaches [21]. Carbon nanotubes/photosensitizer hybrid could supply the nanotubes with many of the photosensitizer’s unique intrinsic properties, e.g., fluorescence and the generation of cytotoxic singlet oxygen, and could overcome most of the shortcomings of classic photosensitizers, such as passive targeting and poor aqueous solubility [22,23]. Recently, we reported a nanoprobe based on the polyehylene-glycol modified single-walled carbon nanotubes (SWCNTs) and photosensitizer with highly stable structure and low dark toxicity [24]. However, further selectivity and efficacy studies are needed to validate the potential of carbon nanotube-based materials for the delivery of photosensitizers. The limited selectivity and efficacy are still widely accepted problems for photosensitizers associated with PDT treatments [25].

In this context, we developed a novel cancer targeting theranostic nanoprobe based on SWCNTs modified with a natural biopolymer chitosan, a photosensitizing drug pyropheophorbide a, a tumor-homing molecule folic acid, and a fluorescence imaging agent fluorescein isothiocyanate. The as-prepared nanoprobe can not only improve the selectivity and solubility of photosensitizers, but also can be used for effective cancer cell targeting imaging and fluorescence imaging-guided photodynamic therapy. The nanoprobe can exhibit the benefits of PDT, nanosized drug delivery SWCNTs, and natural biopolymer chitosan. Advantageously, the nanoprobe has dual-selectivity through both folic acid active targeting and light irradiation selectively on the tumor.

## 2. Results and Discussion

### 2.1. Preparation of PPa/FITC-SWCNT-FA

**Figure 1:**
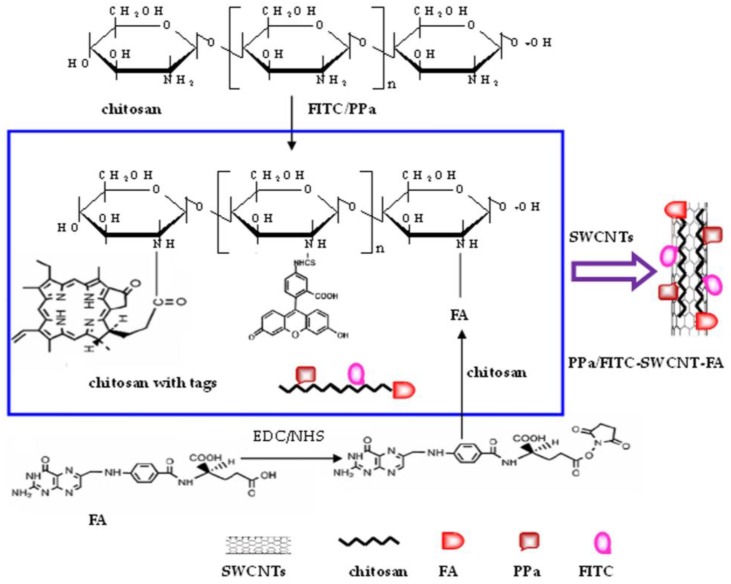
Synthetic scheme of the targeting theranostic nanoprobe based on folic acid (FA), pyropheophorbide a (PPa), fluorescein isothiocyanate (FITC), and chitosan (CHIT)-comodified single-walled carbon nanotubes (SWCNTs).

A folate receptor-targeting theranostic nanoprobe based on folic acid (FA), pyropheophorbide a (PPa), fluorescein isothiocyanate (FITC), and chitosan (CHIT)-comodified single-walled carbon nanotubes (SWCNTs) was developed, as shown in Figure 1. The surface of chitosan was first grafted with FA, PPa, and FITC to form functional chitosan which was denoted as CHIT-FA, CHIT-FITC, and CHIT-PPa, respectively. FA and PPa were bounded to chitosan by the covalently linkage of the carboxyl group of FA and PPa with the amino group of chitosan, with 1-ethyl-3-(3-dimethylaminopropyl) carbodiimide hydrochloride and *N*-hydroxysuccinimide to accelerate the reaction. FITC was used to attach a fluorescent label to chitosan via the covalently linkage of the isothiocyanate group of FITC with the amino group of chitosan. Then SWCNTs were sonicated in aqueous solution of CHIT-FA + CHIT-FITC + CHIT-PPa for 6 h in dark at room temperature. Finally, the mixture of SWCNTs and functional chitosan was centrifugated to remove aggregated SWCNTs, and filtrated to remove unbound CHIT-FA, CHIT-FITC and CHIT-PPa in order to form the nanoprobe PPa/FITC-SWCNT-FA. The as-prepared PPa/FITC-SWCNT-FA was kept at 4 °C for storage. The same procedure has been followed for the preparation of the control probe, FITC-SWCNT-FA, and PPa-SWCNT-FA. Here, chitosan was chosen as it is a natural biocompatible biopolymer with favorable physicochemical properties and can potentially enhance the therapeutic effect [26,27]. Chitosan not only provides an appropriate biological interface to bind FA, PPa and FITC, but also provides a means to effectively disperse the as-prepared PPa/FITC-SWCNT-FA in aqueous solution. SWCNTs was used as drug delivery carriers, PPa as a photosensitizing drug, FA as a tumor-homing molecule, and FITC as a fluorescence imaging agent, affording the resulting probe used for cancer cell targeting imaging and fluorescence imaging-guided photodynamic therapy.

The as-prepared PPa/FITC-SWCNT-FA nanoprobe has three major functions: targeting, imaging and therapy. PPa/FITC-SWCNT-FA has dual targeting, through both folic acid active targeting and light irradiation selectively on the tumor, which could be selectively internalized by targeted cells via the folate receptor-mediated endocytosis. The imaging ability of PPa/FITC-SWCNT-FA is based on the fluorescence imaging agent FITC. FITC excited by 488-nm light leads to a fluorescence emission maximum around 520 nm. Then FITC fluorescence is used for tumor cell fluorescence imaging. The therapy ability of PPa/FITC-SWCNT-FA is based on PPa, a photosensitizing drug. PPa can be activated by 635-nm light to generate cytotoxic singlet oxygen (^1^O_2_) with surrounding oxygen molecules, resulting in tumor cells necrosis and apoptosis.

### 2.2. Morphology Characterization and UV-Vis Analysis

**Figure 2:**
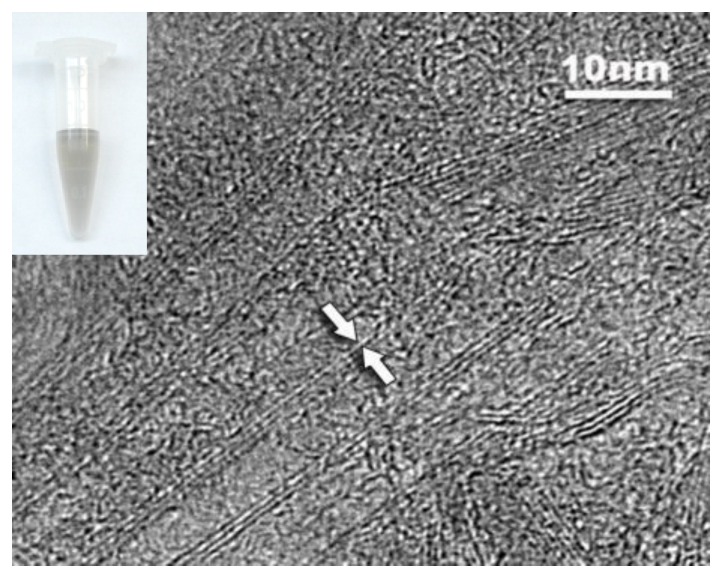
A typical transmission electron microscope image of SWCNTs and a photography of SWCNTs solution (inset).

SWCNTs were solubilized in the aqueous chitosan solution, evidenced by transmission electron microscope (TEM) measurement and the homogeneous black color of SWCNTs solution, as shown in Figure 2. The SWCNTs sample was observed by dropping 5 µL of the solution on the top of a TEM grid. The results show that SWCNTs can exist both individually and in small bundles, indicating a satisfactory dispersibility. SWCNTs solution was stable for at least two weeks at room temperature without visible flocculation, which should be attributed to non-covalent adsorption between chitosan chains and SWCNTs. Our previously published work indicated that chitosan is potential as an ideal candidate to modify SWCNTs, and the chitosan-wrapped SWCNTs are a potential device for drug delivery [28]. Compared to other synthetic polymers, chitosan is a safe substance for the human organism [29,30]. The intrinsic properties of chitosan make it one of the most preferred biomaterials for developing drug and cell delivery matrices [31,32].

**Figure 3:**
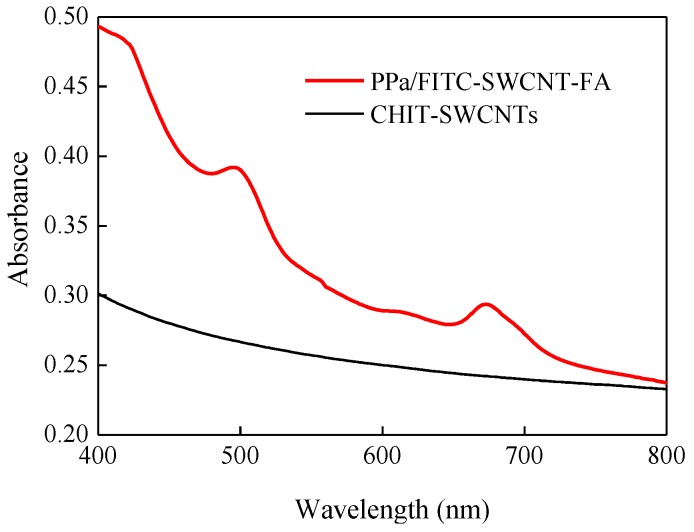
Absorbance spectra of PPa/FITC-SWCNT-FA and plain CHIT-SWCNTs.

In order to validate concentration for PPa and FITC on the SWCNTs, UV-Vis measurement of PPa/FITC-SWCNT-FA was carried out. The concentration-dependent absorption spectra of PPa and PPa absorbance calibration curve at 673 nm were shown in Figure S1 and Figure S2 was the absorption spectra of FITC at different concentration and FITC absorbance calibration curve at 495 nm. The standard curve described by the following typical equation: *Y* = −0.0029 + 0.0114*C*_PPa_ (µM, *R*^2^ = 0.9957) and *Y* = −0.0064 + 0.0419*C*_FITC_ (µM, *R*^2^ = 0.9924). Figure 3 shows UV-Vis absorbance spectra of PPa/FITC-SWCNT-FA (red curve) and plain CHIT-SWCNTs (dark curve). PPa/FITC-SWCNT-FA exhibits two absorbance peaks at 495 nm and 673 nm on top of the plain CHIT-SWCNTs absorption spectrum. Note that unbound CHIT-FITC and CHIT-PPa have been thoroughly removed from PPa/FITC-SWCNT-FA solution. The absorbance peak of PPa/FITC-SWCNT-FA at 495 nm corresponds to the characteristic peak of FITC, and the peak at 673 nm corresponds to the characteristic peak of PPa. The concentrations of PPa and FITC on the SWCNTs were determined as about 4.99 µM and 0.98 µM by UV-Vis measurement via the standard curve method, respectively. FITC loaded on the SWCNTs can provide a rapid and sensitive means to character cellular uptake of PPa/FITC-SWCNT-FA, and can be used for fluorescence imaging-guided PDT therapy.

### 2.3. Targeting Specificity Imaging

**Figure 4:**
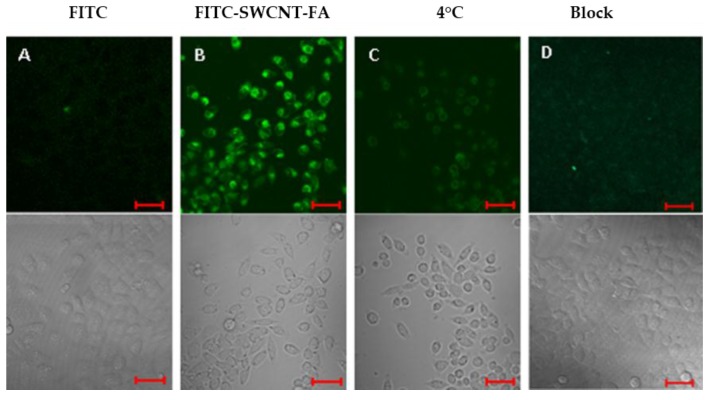
Optical microscopy images of HeLa cells incubated with FITC (**A**); FITC-SWCNT-FA at 37 °C (**B**) or 4 °C (**C**); and FITC-SWCNT-FA in the presence of free FA (denoted as Block, **D**). Confocal fluorescence images (**top**) and transmittance images (**bottom**). Scale bar, 50 μm.

To investigate the tumor targeting and fluorescent imaging capability of the probe, FITC-SWCNT-FA and FITC were incubated with HeLa cells cultured in FA-free Dulbecco’s modified Eagle’s medium (DMEM) to ensure the overexpression of folate receptors on the cell surfaces. Then the FITC fluorescence emissions of fixed cells were obtained by confocal microscopy, as shown in Figure 4. Here, FITC was excited at 488 nm with an Ar-ion laser and the fluorescence emission was recorded through a 500–530 nm band-pass filter. The results indicate FITC itself cannot transport inside HeLa cells because there is no fluorescence signal inside the cells (Figure 4A). The green color corresponds to the fluorescence emission of FITC on FITC-SWCNT-FA, confirming the cellular uptake of FITC-SWCNT-FA by HeLa cells (Figure 4B).

In order to further confirm the target specificity, fluorescent images of HeLa cells treated with FITC-SWCNT-FA at 4 °C or blocking experiments by adding FITC-SWCNT-FA with free FA (1 mM) to the HeLa cells were conducted as the control groups. The fluorescence level detected either in the low temperature control group (Figure 4C) or in the free FA blocking control group (Figure 4D) was significantly lower than that of the experiment group (Figure 4B). This suggests that the cellular uptake of FITC-SWCNT-FA can be effectively blocked either by low temperature or by free FA, confirming the folate receptor-mediated endocytosis. The observed cellular uptake of the FITC-SWCNT-FA is similar to those of targeted delivery of conjugates of FA with nanomaterials to folate receptor-overexpressed tumor cells [33,34]. This active targeting can help the probe to overcome biological barriers, decrease the residual toxicity, and increase the therapeutic effect [35]. Advantageously, the proposed nanoprobe can offer the possibility of dual-selectivity in cancer therapy, through both FA active targeting and light irradiation selectively on the tumor.

### 2.4. Fluorescence Imaging-Guided Photodynamic Therapy

**Figure 5:**
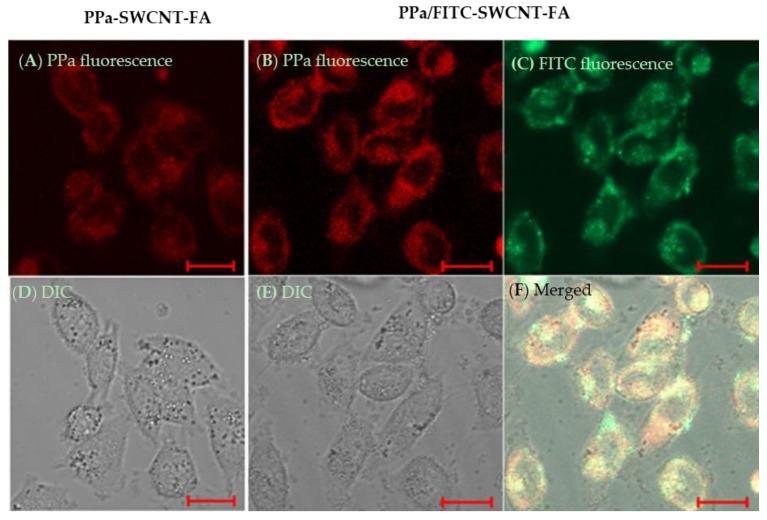
Optical microscopy images of Hela cells treated with PPa-SWCNT-FA (**A**,**D**); and PPa/FITC-SWCNT-FA (**B**,**C**,**E**,**F**); Confocal fluorescence images (**A**,**B**,**C**,**F**) and transmittance images (**D**,**E**). FITC excited at 488 nm. PPa excited at 633 nm. Scale bar, 20 μm.

The PPa/FITC-SWCNT-FA can be utilized for fluorescence imaging-guided cancer photodynamic therapy using FITC fluorescence as the imaging fluorescence, and through the generation of cytotoxic singlet oxygen to destroy cancer cells upon the illumination of a photosensitizer PPa at a 635 nm laser. Since the action radius of cytotoxic singlet oxygen is less than 20 nm, so the cellular uptake of photosensitizer is of great significance when using photodynamic therapy in cancer cells [9]. Cellular uptake of photosensitizer was visualized, as shown in Figure 5. The red fluorescence is clear inside HeLa cells treated with PPa-SWCNT-FA. The red fluorescence corresponds to the fluorescence emission of PPa on the PPa-SWCNT-FA, confirming the efficient cellular uptake of photosensitizer. Importantly, confocal images of HeLa cells treated with PPa/FITC-SWCNT-FA show that fluorescence emissions of PPa coincide with that of FITC, confirming that the PPa/FITC-SWCNT-FA can be used for cancer cell targeting imaging and fluorescence-guided photodynamic therapy.

PPa/FITC-SWCNT-FA cytotoxicity on HeLa cells was investigated using CCK8 assay. HeLa cells were treated with PPa/FITC-SWCNT-FA and laser, free PPa and laser, laser alone, or PPa/FITC-SWCNT-FA alone, respectively. HeLa cells were incubated with PPa/FITC-SWCNT-FA nanoprobe or free PPa for 2 h at 37 °C, and rinsed with PBS. After incubation, HeLa cells were irradiated with 5 J/cm^2^ light dose using a 635 nm laser at a power density of 20 mW/cm^2^. Hela cells treated with laser alone or PPa/FITC-SWCNT-FA alone showed a slight viability decrease of about 7%–13%. The viability drop of Hela cells treated with free PPa and laser was about 12%–31%, while the viability drop of HeLa cells treated with PPa/FITC-SWCNT-FA and laser was about 60%–74%, indicating satisfying biocompatibility and antitumor activity.

**Figure 6:**
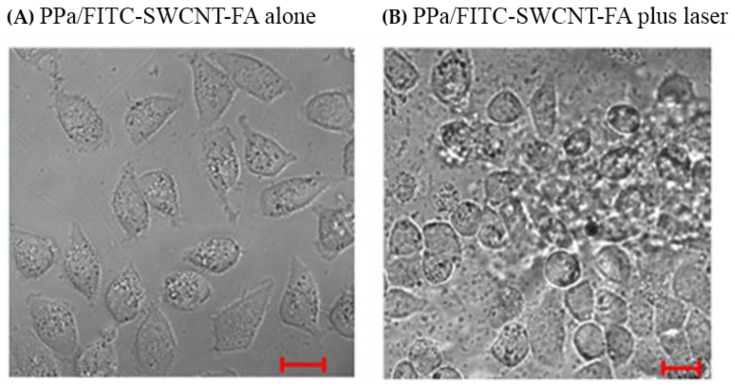
Typical optical microscopy images of HeLa cells treated with PPa/FITC-SWCNT-FA alone (**A**) and PPa/FITC-SWCNT-FA and laser (**B**). Scale bar, 20 μm.

To further investigate the destructive effect of PPa/FITC-SWCNT-FA under laser irradiation on HeLa cells, we detected cell morphology. Figure 6 shows the typical optical microscopy images of HeLa cells treated with PPa/FITC-SWCNT-FA alone (A), and PPa/FITC-SWCNT-FA plus laser (B). HeLa cells growing in 35-mm Petri dishes were incubated with PPa/FITC-SWCNT-FA solution for 2 h, rinsed with PBS, replaced with fresh cell media, and exposed to with or without a 635 nm laser beam at the same light dose as CCK8 assay. HeLa cells treated with PPa/FITC-SWCNT-FA alone remain intact. In contrast, HeLa cells treated with PPa/FITC-SWCNT-FA plus laser showed drastic cell morphology change, which is in close agreement with previous work in literature [36]. Furthermore, photodynamic therapy might increase the immunogenicity of dead tumor cells by exposing or creating new antigens, and by inducing heat-shock proteins that increase the efficiency of antigen cross-presentation to form more effective tumor-specific cytotoxic T cells [37]. These results indicated that the as-prepared PPa/FITC-SWCNT-FA has potential as a novel targeting theranostic nanoprobe for effective cancer cell targeting imaging and fluorescence imaging-guided photodynamic therapy.

## 3. Materials and Methods

### 3.1. Materials

Single-walled carbon nanotubes (SWCNTs) were obtained from Chinese Academy of Sciences (Chengdu, China). Pyropheophorbide a (PPa) was purchased from J&K Scientific Ltd. (Beijing, China). Fluorescein isothiocyanate (FITC), 1-ethyl-3-(3-(dimethylamino)-propyl) carbodiimide hydrochloride (EDC), *N*-hydroxysulphonosuccinimide (NHS), folic acid (FA), dimethylsulfoxide (DMSO), penicillin, and streptomycin were obtained from Sigma-Aldrich (St. Louis, MO, USA). Cell Counting Kit-8 (CCK8) was obtained from Dojindo laboratories (Shanghai, China), and fetal bovine serum from Zhejiang Tianhang Biotechnology Co., Ltd. (Hangzhou, China). Water-soluble chitosan (CHIT) was purchased from Zhejiang Golden Shell Pharmaceutical Co., Ltd. (Taizhou, China). Dulbecco’s modified Eagle’s medium (DMEM) was obtained from Gibco (Gaithersburg, MD, USA). Since all the reagents were of analytical grade, they were used without further purification.

### 3.2. Apparatus and Instruments

The absorption spectra were obtained using a UV-Vis spectrometer (Lambda 35, Perkin-Elmer, Norwalk, CT, USA). The morphology and size of SWCNTs was observed on a JEM-2010HR transmission electron microscope (JEOL, Tokyo, Japan). The fluorescence imaging and optical microscopy images of HeLa cells were conducted at a Zeiss LSM-510 Confocal Microsope (Zeiss, Jena, Germany), and the absorbance value at 450 nm of each well at 96-well culture plates for CCK8 was read with a microplate reader infinite M200 (TECAN, Mannedorf, Switzerland).

### 3.3. Preparation of Functional SWCNTs

*Synthesis of CHIT-FA and CHIT-PPa.* FA was dissolved in DMSO and activated by EDC/NHS to afford FA-NHS (molar ratio, FA:EDC:NHS = 1:1.2:1.2) at room temperature for overnight. Then chitosan was mixed with FA-NHS (mass ratio 5:1), and incubated for 6 h in dark. After reaction, the solution was filtrated using 3500 Da filters (Millipore, Billerica, MA, USA) to remove excess FA-NHS, EDC, and NHS, and subsequently freeze-dried in a lyophilizer (Ilshin, Dongducheon, Korea) to obtain CHIT-FA. The synthesis of CHIT-PPa was prepared by the same procedure.

*Synthesis of CHIT-FITC.* 100 µL FITC (1 mg/mL) dissolved in DMSO was added to 1 mL of chitosan (1 mg/mL) in PBS (pH 7.4). The mixture was stirred at room temperature for overnight in dark, and filtrated using 3500 Da filters (Millipore) to remove excess FITC, and subsequently freeze-dried in a lyophilizer to obtain CHIT-FITC.

*Functionalization of SWCNTs.* SWCNTs were sonicated in aqueous solution of CHIT-FA + CHIT-FITC + CHIT-PPa (abbreviated as PPa/FITC-SWCNT-FA) for 6 h in dark at room temperature, and centrifugated at 5000× *g* for 10 min to remove aggregated SWCNTs (2 mg of SWCNTs: 1 mg of CHIT-FA: 1 mg of CHIT-FITC: 1 mg of CHIT-PPA: 2 mL of PBS). Then the supernatant was collected and filtrated through 100 KDa filters (Millipore) to remove unbound free CHIT-FA, CHIT-FITC and CHIT-PPa. Finally, the functional SWCNTs, PPa/FITC-SWCNT-FA, were obtained, and kept at 4 °C for storage. The same procedure has been followed for the preparation of CHIT-FA + CHIT-FITC modified SWCNTs (abbreviated as FITC-SWCNT-FA), CHIT-FA + CHIT-PPa modified SWCNTs (abbreviated as PPa-SWCNT-FA).

### 3.4. Cell Culture

HeLa cells were cultured in Dulbecco’s modified Eagle’s medium (DMEM). The cell culture mediums were supplemented with 10% fetal bovine serum, penicillin (100 units/mL) and streptomycin (100 µg/mL) in 5% CO_2_, 95% air at 37 °C in humidified incubator.

### 3.5. Morphology Characterization and UV-Vis Analysis

SWCNT sample was observed by a JEM-2010HR transmission electron microscope (TEM) by dropping 5 μL of the solution on the top of a TEM grid. The optical absorbance characteristics of functional SWCNTs were investigated by visible absorption spectra (Lambda-35 UV-Vis spectrophotometer, Perkin-Elmer, Norwalk, CT, USA).

### 3.6. Confocal Microscopy

HeLa cells were seeded into 35-mm Petri dishes and incubated for 24 h. The cell culture medium was removed and the DMEM containing the functional SWCNTs was added into each well, followed by incubation at 37 °C for 2 h. After incubation, HeLa cells were washed by changing the fresh DMEM and then characterized by confocal microscopy with a commercial laser scanning microscope (LSM 510/ConfoCor 2) combination system (Zeiss, Jena, Germany) equipped with a Plan-Neofluar 40×/1.3 NA Oil DIC objective. The excitation wavelength and detection filter settings for each of the fluorescence indicators were as follows. FITC was excited at 488 nm with an Ar-ion laser, and the fluorescence emission was recorded through a 500–530 nm band-pass filter. PPa was excited at 633 nm with a He-Ne laser, and the fluorescence emission was recorded through a 650 nm long-pass filter.

### 3.7. Cell Cytotoxicity Assay Using Cell Counting Kit-8 (CCK8)

Cell cytotoxicity was evaluated by determining the viability of HeLa cells with a colorimetric tetrazolium salt-based assay, CCK8. HeLa cells were seeded in 96-well culture plates at 1 × 10^4^ cells per well. After being cultured for 24 h, the cell culture medium was removed and the DMEM containing the functional SWCNTs was added into each well, followed by incubation at 37 °C for 2 h. The cells were washed to remove the unbound functional SWCNTs and irradiated with 5 J/cm^2^ light dose using a 635 nm laser at a power density of 20 mW/cm^2^. Cell cytotoxicity was assessed 24 h after the laser irradiation with CCK8. The absorbance of each well at 450 nm was measured by Infinite M200 (TECAN, Mannedorf, Switzerland) to determine the cell viability.

## 4. Conclusions

We synthesized a targeting theranostic nanoprobe based on functional carbon nanotubes-chitosan composites (PPa/FITC-SWCNT-FA), and examined the potential application for cancer cell targeting imaging and fluorescence imaging-guided PDT by assessing their water-dispersible, targeting, and antitumor activity. The as-prepared PPa/FITC-SWCNT-FA was stable for at least two weeks at room temperature with a good dispersibility. Confocal fluorescence imaging experiments confirmed that the PPa/FITC-SWCNT-FA can not only specifically target the cancer cells which overexpress folate-receptors, but also has the ability to effective tumor cell targeting imaging. Cell cytotoxicity assay showed the dark toxicity is about 7%–13%, and the phototoxicity is about 60%–74%, indicating a satisfying biocompatibility and antitumor activity. Combining the intrinsic properties of PDT and chitosan-carbon nanotubes composite, and the dual targeting capabilities, through both folic acid active targeting and light irradiation selectively on the tumor, the PPa/FITC-SWCNT-FA has potentials as a theranostic agent for application in cancer therapy. However, there are still plenty of spaces for optimization studies of the PPa/FITC-SWCNT-FA preparation, quantitative fluorescence imaging, a decrease of dark toxicity, and an increase of phototoxicity, which are further pursuits of our efforts.

## Supplementary Materials

The following are available online at http://www.mdpi.com/2079-4991/6/11/216/s1.

## Abbreviations

SWCNTs	Single-walled carbon nanotubes
CHIT	Chitosan
FA	Folic acid
FITC	Fluorescein isothiocyanate
PPa	Pyropheophorbide a
PDT	Photodynamic therapy
EDC	1-ethyl-3-(3-dimethylaminopropyl) carbodiimide hydrochloride
NHS	*N*-hydroxysuccinimide
TEM	Transmission electron microscope
DMEM	Dulbecco’s modified Eagle’s medium
DMSO	Dimethylsulfoxide
CCK8	Cell Counting Kit-8
