# Supplementary material for: A Targeted Nanoprobe Based on Carbon Nanotubes-Natural Biopolymer Chitosan Composites
Source: Nanomaterials (Basel). 2016 Nov 17;6(11):216. doi: 10.3390/nano6110216 (PMC5245750; doi:10.3390/nano6110216)
Supplement: Supplementary file 1 [file nanomaterials-06-00216-s001.pdf]

Supplementary Materials: A Targeted Theranostic Nanoprobe Based on Carbon Nanotubes-Natural Biopolymer Chitosan Composites

Baoyan Wu and Na Zhao

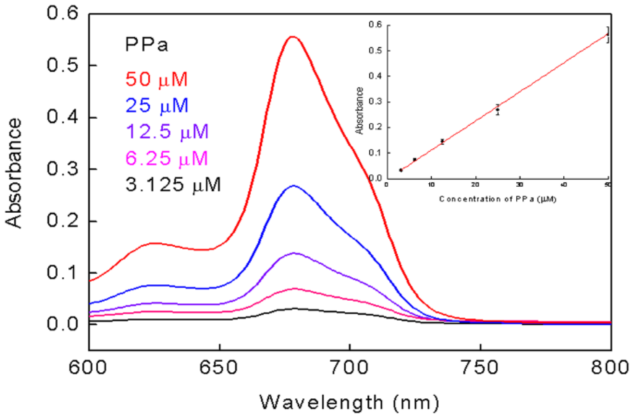

Figure S1. The absorption spectra of PPa with different concentrations. Inset: The standard curve for PPa absorbance value at 673 nm. Data represent mean values ± standard deviation, *n* = 3.

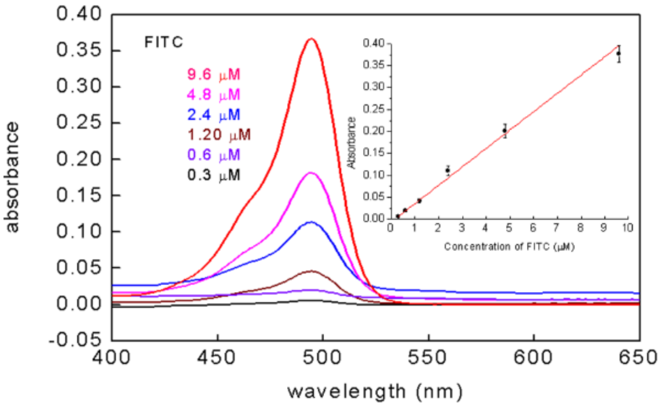

Figure S2. The absorption spectra of FITC with different concentrations. Inset: The standard curve for FITC absorbance value at 495 nm. Data represent mean values ± standard deviation, *n* = 3.

© 2016 by the authors. Submitted for possible open access publication under the terms and conditions of the Creative Commons Attribution (CC-BY) license (http://creativecommons.org/licenses/by/4.0/).
